# A Retinol Isotope Dilution Equation Predicts Both Group and Individual Total Body Vitamin A Stores in Adults Based on Data from an Early Postdosing Blood Sample^1^,^2^,^3^

**DOI:** 10.3945/jn.116.233676

**Published:** 2016-08-10

**Authors:** Michael H Green, Jennifer Lynn Ford, Joanne Balmer Green, Philip Berry, Alan V Boddy, Anthony Oxley, Georg Lietz

**Affiliations:** 4 Department of Nutritional Sciences, The Pennsylvania State University, University Park, PA; and; 5 Northern Institute for Cancer Research and; 6 Human Nutrition Research Centre, Newcastle University, Newcastle Upon Tyne, United Kingdom

**Keywords:** humans, interindividual variation, liver vitamin A, retinol isotope dilution technique, vitamin A status, vitamin A stores, WinSAAM

## Abstract

**Background:** Retinol isotope dilution (RID) is used to determine vitamin A total body stores (TBS) after an oral dose of a vitamin A stable isotope. The generally accepted prediction equation proposed by Olson’s group in 1989 (Furr et al. Am J Clin Nutr 1989;49:713–6) includes factors related to dose absorption and retention, isotope equilibration in plasma compared with stores, catabolism during the mixing period, and the optimal time for measuring plasma isotope enrichment.

**Objectives:** The objectives were *1*) to develop a modified RID equation and identify an earlier sampling time for predicting TBS and *2*) to improve prediction in individuals as well as groups.

**Methods:** To develop a modified RID equation, we used results of model-based compartmental analysis [the Simulation, Analysis and Modeling software (WinSAAM version 3.0.8; http://www.WinSAAM.org)] of plasma [^13^C_10_]retinol kinetic data from 32 previously studied, healthy young adults of European ancestry who had moderate vitamin A intakes and who ingested 2.95 μmol [^13^C_10_]retinyl acetate.

**Results:** We examined the time dependence of factors in the prediction equation related to absorption/retention (*Fa*) and isotope equilibration (*S*) and determined that 4 or 5 d postdosing was the optimal sampling time. TBS calculated by the equation TBS = *Fa* x *S* x (1/SA_p_), where SA_p_ is plasma retinol specific activity (fraction of dose/μmol), were highly correlated with model-predicted TBS (*r* = 0.95 and 0.96 for 4 and 5 d, respectively; *P* < 0.001); predictions for individuals were also highly correlated (*R*_s_ = 0.94 and 0.94; *P* < 0.001).

**Conclusion:** The equation TBS ≈ 0.5 × (1/SA_p_) accurately predicted vitamin A TBS in this group of 32 healthy young adults and its individual members with the use of data from 1 blood sample taken 4 d after isotope administration.

See corresponding commentary on page 1929 and article on page 2129.

## Introduction

There continues to be keen interest among nutritionists and public health experts in refining methods for assessing vitamin A status (i.e., stores) in the field. The retinol isotope dilution (RID^8^) technique, recently described (1) as the method that provides “the most sensitive and accessible quantitative assessment of body retinol stores across a wide range of vitamin A status,” has been used extensively but, as currently applied, is not fully satisfactory to researchers (see below).

The most commonly used form of the RID prediction equation was developed in the late 1980s in Olson’s laboratory (2), based on earlier work on the use of isotope dilution to estimate liver vitamin A concentrations in humans and in animal models (3, 4). Furr et al. (2) in Olson’s laboratory obtained liver biopsy samples from 11 adults, determined liver vitamin A concentrations, and extrapolated those values to estimate total liver vitamin A content. Within 1 wk of biopsy, a large dose (45 mg) of tetradeuterated retinyl acetate in corn oil was administered orally to each patient, and a blood sample was collected 10–47 d after tracer dosing for the estimation of total liver vitamin A reserve by using the isotope dilution equation shown below (Equation *1*). When predictions of the equation were compared with estimates based on liver biopsies for 10 of the subjects, there was a linear correlation (*r* = 0.88):
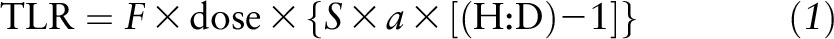
where TLR was the total liver reserve of vitamin A, *F* was the fraction of the orally administered dose of deuterated vitamin A that was absorbed and retained, *S* was the ratio of specific activity of retinol in plasma to that in liver after equilibration of the dose with body stores, *a* was a correction for fraction of the absorbed dose lost via catabolism, H:D was the measured hydrogen-to-deuterium ratio in plasma retinol after mixing was complete, and −1 adjusted for the mass of vitamin A added to stores by the administered dose. Although the parameter calculated by Equation *1* was called TLR by Furr et al. (2), it is now generally accepted that what is actually determined is vitamin A total body stores (TBS), because all of the normal body’s vitamin A is in dynamic equilibrium (i.e., it is fully exchangeable) as originally shown by Rietz et al. (5) and subsequently confirmed by Green and Green (6). Thus, when a vitamin A tracer is administered, it will mix or interfuse with vitamin A in all of the body’s exchangeable pools, and the dilution of the tracer in a plasma sample obtained at an appropriate time after dose administration will reflect TBS of the vitamin.

Since publication of the Olson equation in 1989, it has been applied by a number of research groups to estimate vitamin A status [see references in (7) as well as references 8 and 9], and the equation has been extensively discussed (7, 10, 11). In one study, Haskell et al. (12) showed that, compared with estimates based on liver biopsies, the equation did a good job of predicting mean TBS in a group of 31 Bangladeshi subjects with low liver vitamin A concentrations; however, the prediction for individual subjects was not very good.

Here we present a different version of the original RID equation, that to our knowledge addresses some of the limitations in the original method, including the long time (∼3 wk) between dosing and sampling as well as the inability to estimate TBS in individual subjects. The current article was preceded by preliminary work described in reference 13 in which we had begun to explore the values assigned to the factors in the original equation as well as conditions that may affect RID results. The new equation presented here uses data collected at 4 or 5 d after dose administration, and it provides accurate estimates of TBS in individuals. To develop the new equation, we used results from compartmental analysis of vitamin A kinetics in a group of healthy young adults of European ancestry (14); this allowed us to determine individual subject values for the coefficients in Equation *1* and to validate the new equation by comparing its prediction to TBS from the compartmental model. The use of data collected sooner after dose administration not only provides better discrimination between different vitamin A statuses in both groups and individuals but may also increase subject availability/compliance and ensure a lower likelihood of changes in health status during the test (e.g., development of infections).

## Methods

### 

#### Subjects, design, and compartmental analysis.

References 14 and 15 contain details related to subject characteristics, informed consent, stable isotope doses, and analyses. The current study is based on data from 32 healthy young-adult subjects (see Results) whose results for plasma [^13^C_10_]retinol kinetics after the oral ingestion of [^13^C_10_]retinyl acetate (2.95 μmol) were suitable for steady state model-based compartmental analysis. As described in reference 14, 11 blood samples were collected from each subject from baseline until 14 d after dose administration; plasma [^13^C_10_]retinol was determined by LC–tandem mass spectrometry (LC-MS/MS) and plasma [^12^C]retinol was determined by HPLC.

**FIGURE 1:**
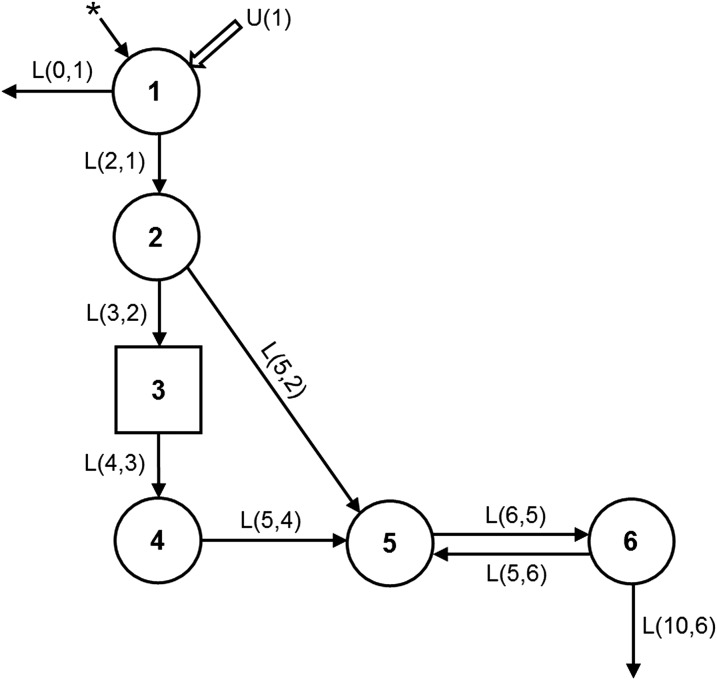
Compartmental model for retinol kinetics in humans. Circles represent compartments; component 3, shown as a rectangle, is a delay element, and interconnectivities between compartments [L(I,J)s] are fractional transfer coefficients, or the fraction of retinol in compartment J that is transferred to compartment I each day. Compartments 1–4 (including component 3) correspond to vitamin A digestion and absorption, chylomicron production and metabolism, liver uptake of chylomicron remnant retinyl esters, and hepatic processing of retinol. Compartment 5 represents plasma retinol bound to retinol-binding protein and transthyretin; this retinol exchanges with vitamin A in 1 extravascular pool (compartment 6), which includes liver vitamin A stores. The asterisk (*) represents the site of input of orally administered [^13^C_10_]retinyl acetate, and U(1) represents dietary vitamin A input. L(I,J), fractional transfer coefficient. Adapted from reference 19 with permission.

Data on the fraction of the oral dose (FD) in plasma [^13^C_10_]retinol compared with time for each subject were analyzed by model-based compartmental analysis with the use of WinSAAM version 3.0.8, the Windows version of the Simulation, Analysis and Modeling software (WinSAAM; http://www.WinSAAM.org) (16–18), in light of a 6-component model (**Figure 1**) developed by Cifelli et al. (19) as described in reference 14. Data for each subject were fit to the model, and values for the fractional transfer coefficients [L(I,J)s; or the fraction of retinol in compartment J that is transferred to compartment I each day] were determined by using weighted nonlinear regression analysis. Then, the estimated plasma retinol pool size [mean concentration × estimated plasma volume; M(5) or M_p_] was used in a steady state solution to predict compartment pool sizes [M(I)] and, in particular, vitamin A TBS [M(6) or M_s_; the mass of vitamin A in compartment 6 (Figure 1)]. The model was also used to simulate values for the coefficients in Equation *1* over time, extending predictions beyond the experimental time to 28 d.

#### Statistical methods.

Data were managed by using Microsoft Excel; graphing and least-squares regression analyses were conducted by KaleidaGraph (version 4.1; Synergy Software) and GraphPad Prism (version 6; GraphPad Software). Pearson’s correlation coefficients (*r*), Spearman rank correlation coefficients (*R*_s_), the coefficient of determination (*R*^2^), and CV% were determined. *P* < 0.05 was considered significant.

## Results

### 

#### Subject characteristics.

Table 1 in reference 14 presents information on 30 of the 32 participants; 2 other subjects from reference 15 were included here so that the group was composed of 15 men and 17 women with a mean ± SD age of 24 ± 4 y, a mean BMI (kg/m^2^) of 23 ± 2, and moderate intakes of preformed vitamin A (444 ± 481 μg/d) as determined by an FFQ. The mean plasma retinol concentration during the study for all subjects was 1.52 ± 0.27 μmol/L.

#### Preliminary considerations.

To develop a modified RID equation that would provide more accurate predictions of TBS in individual subjects, we began by reviewing previous work and examining the assumptions in the original RID equation (2) (Equation *1* above). We concluded the following: *1*) it would be ideal to have an equation that accurately estimated TBS on the basis of data collected during the first week after isotope administration; *2*) the factor −1 that was included in the original equation to correct calculated TBS for the mass of vitamin A in the dose should be eliminated because the measured variable was H:D, not (H+D)/D, rendering the −1 an error; *3*) we also wanted to express the factor “dose × H:D” more generally because some researchers use [^13^C] for RID studies and because, for vitamin A modeling studies, plasma data are typically expressed as FD. Expressing current data for plasma [^13^C]retinol as an FD, [^12^C]retinol/[^13^C]retinol FD = 1/SA_p_, where SA_p_ is the specific activity of plasma retinol. Finally, we concluded that *4*) factor *a* was already taken into account in factor *F* so *F* was replaced with *Fa* going forward. See Discussion for additional details on points *1* and *4*.

#### Factor Fa.

**FIGURE 2:**
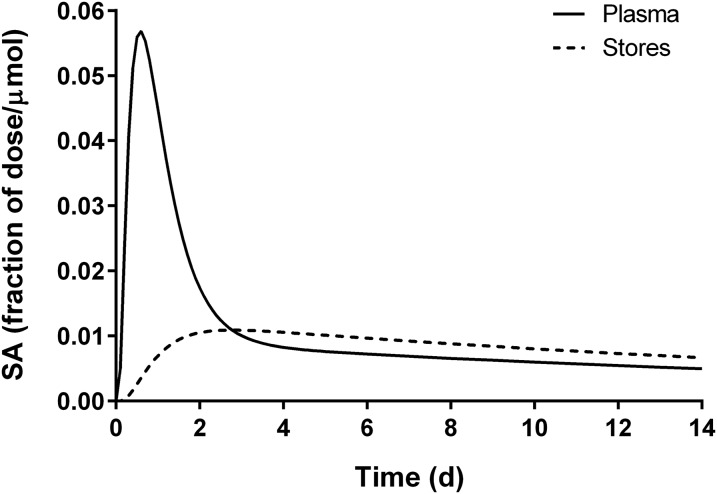
Model-simulated data for retinol specific activity in plasma and in stores vs. time for 1 representative healthy young-adult subject with moderate vitamin A intake. The model is shown in Figure 1. SA, specific activity.

**FIGURE 3:**
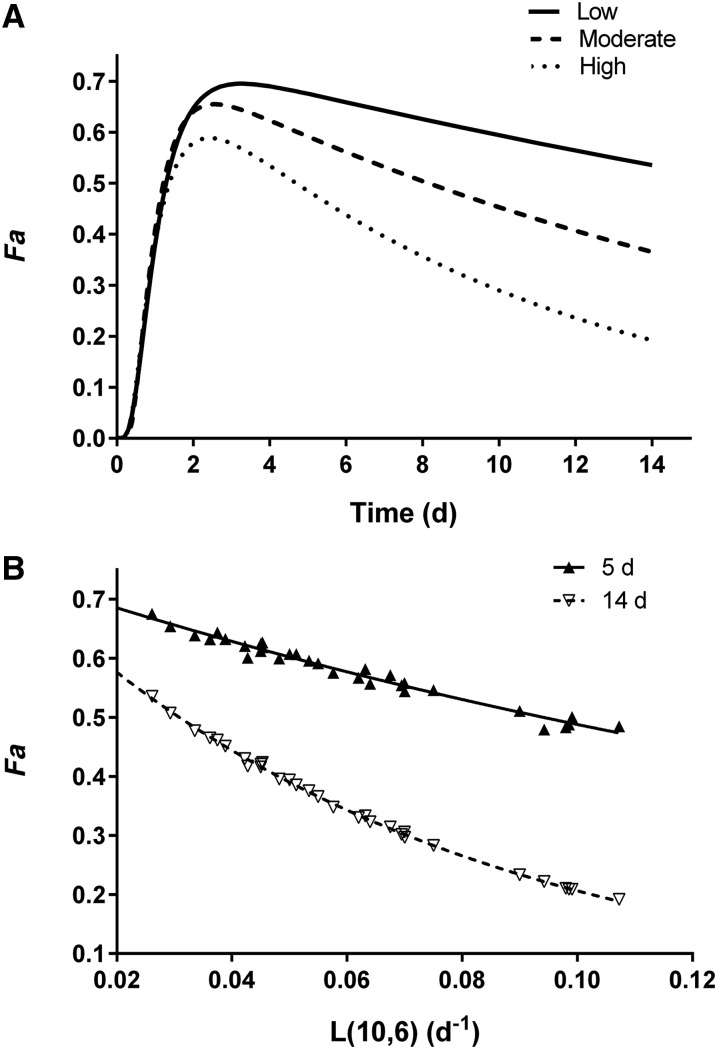
(A) Factor *Fa* vs. time for 3 representative healthy young-adult subjects with moderate vitamin A intakes and either low (2.6%/d), moderate (5.5%/d), or high (10.7%/d) system fractional catabolic rates [L(10,6); Figure 1]. (B) Factor *Fa* compared with model-predicted L(10,6) at 5 and 14 d. Data were fit to the following mono-exponential equations: *y* = 0.74423e^–4.2299^*^x^* for day 5 (*r* = 0.99) and *y* = 0.73988e^–12.782^*^x^* for day 14 (*r* = 1.00), where *y* is *Fa* and *x* is L(10,6). *Fa*, the fraction of the orally administered dose of stable isotope–labeled vitamin A that was absorbed and retained.

Factor *Fa*, the fraction of the administered dose that was absorbed and is still in stores, is equivalent to the fraction of the dose in model compartment 6 (Figure 1). **Figure 2** shows simulated data for plasma and stores vs. time for 1 representative subject. (Note that, although the data in Figure 2 are presented as specific activities, the patterns would be the same for FD because retinol masses were constant over this time period.) The curve for compartment 6 (*Fa*) rises as the isotope begins to appear in stores and it crosses over the curve for compartment 5 at ∼2.7 d, when the tracer reaches a maximum in compartment 6 and tracer input balances output; the curves then become parallel by day 7. The magnitude of factor *Fa* depends on the irreversible loss (catabolism) of vitamin A [i.e., the system fractional catabolic rate (FCR_sys_), which is reflected in the terminal slope of the plasma tracer response curve and as L(10,6) in Figure 1]: a higher FD in stores (i.e., a larger *Fa*) is equivalent to a lower FCR_sys_ and vice versa. **Figure 3A** shows *Fa* vs. time for representative subjects with low, moderate, and high FCR_sys_ (2.6%, 5.5%, and 10.7%/d, respectively). As shown in Figure 3B, L(10,6), the model-predicted fractional loss was highly inversely correlated with *Fa* during the time frame of this study (*r* = 0.99 on day 5 and *r* = 1.00 on day 14; *P* < 0.001 for both days).

#### Factor S.

In the original RID equation (2), *S* was defined as the ratio of specific activity (tracer:tracee) of retinol in plasma to that in liver after equilibration of the dose with body stores. If subjects consume no vitamin A after dose administration, then *S* would equal 1 after the dose equilibrates (isotopic equilibrium). If vitamin A is ingested during the equilibration period, then *S* will be affected by the dilution of the tracer-to-tracee ratio as unlabeled vitamin A (tracee) enters the system from the diet and tracer and tracee are lost by catabolism. For more insight into the most appropriate value for factor *S*, we used modeling results for the current subjects as follows. Because plasma isotope data were expressed as FD, specific activity is the FD divided by mass of the tracee (M). By using that nomenclature, *S* would be written as follows:
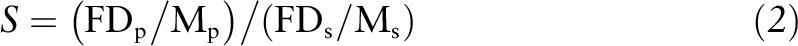
where the subscripts p and s are “plasma” and “stores,” respectively. In the model (Figure 1), compartment 5 is plasma retinol and compartment 6 is extravascular vitamin A stores. When we calculated and simulated data for *S,* we noted large interindividual variations at very early times (i.e., <3 d) after dosing. The value for *S* decreased to 1 at 2.7 d after dosing (range: 1.7–4 d) and reached a plateau (Figure 2) at ∼7 d, when *S* = 0.72.

#### Modified RID equation.

In light of the foregoing, the original RID equation can be restated as follows:
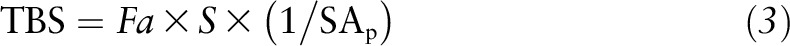
*Fa* and *S* can be assigned values as in past applications of the Olson equation, or the factors can be determined for individual subjects by compartmental modeling as described below. SA_p_ is determined analytically, optimally at a time when the other factors combined (*Fa* × *S*) have the smallest interindividual variation (see later).

#### Model-predicted values for factors Fa and S.

**FIGURE 4:**
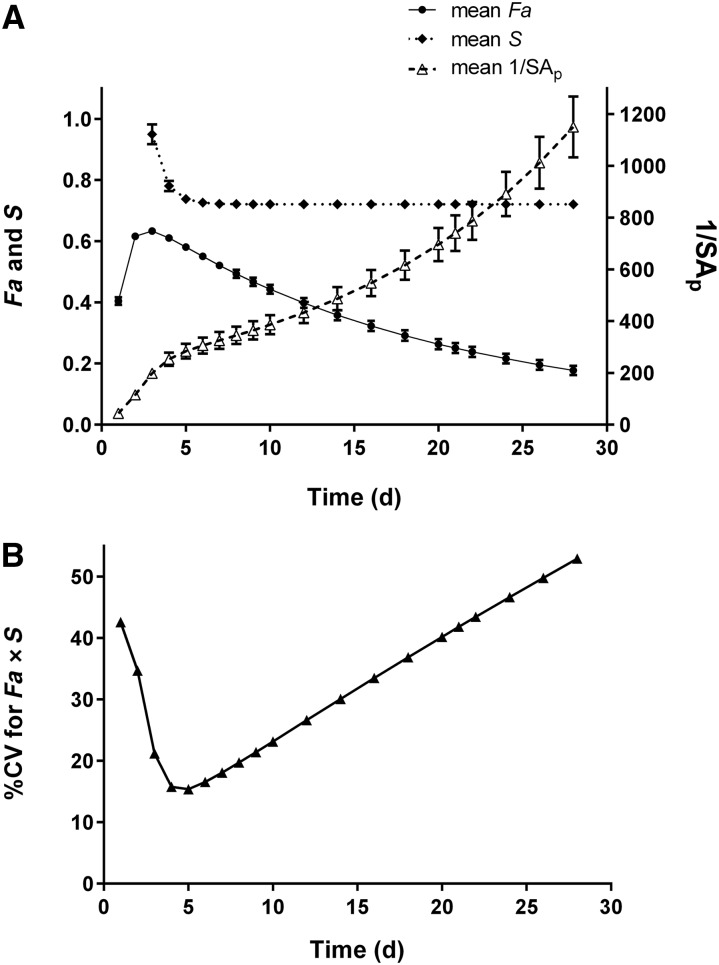
(A) Model-predicted values (means ± SEMs) for *Fa*, *S*, and 1/SA_p_ compared with time based on modeling of plasma retinol response data for 32 healthy young-adult subjects of European ancestry with moderate vitamin A intakes. (B) Variance in *Fa* × *S* over time for the same subjects. *Fa*, the fraction of the orally administered dose of stable isotope–labeled vitamin A that was absorbed and retained; *S*, the ratio of specific activity of retinol in plasma to that in stores; SA_p_, plasma retinol specific activity.

Because the study from which current kinetic data were obtained was short compared with other retinol turnover experiments (19), we simulated our model to 28 d and calculated *Fa*, *S*, and 1/SA_p_ over that time (**Figure 4A**). Mean values for *Fa* peaked at 3 d and decreased substantially by 28 d after dosing; *S* was high right after dosing (7.4 on day 1), and then it decreased to reach a plateau by day 7. For 1/SA_p_, simulation indicated that the value increased steadily over the 28 d, because plasma retinol is constant whereas tracer concentration decreases due to mixing with stores, catabolism of labeled retinol, and intake of unlabeled (dietary) retinol. The CVs for *Fa* × *S* (Figure 4B) decreased from 42% at day 1 to 21% at day 3 and 15% at days 4 and 5; the CV then linearly increased to 53% at day 28, indicating that 4 or 5 d after dosing was the ideal time to most accurately predict both individual and group mean values for TBS. Mean values for the factors at days 4 and 5 were 0.61 and 0.58 for *Fa*, 0.78 and 0.74 for *S*, and 0.48 and 0.43 for *Fa* × *S*, respectively.

#### Using model-predicted Fa × S to calculate TBS for the population and its individuals.

When the mean value for *Fa* × *S* on day 4 and each individual’s 1/SA_p_ were used in Equation *3*, TBS were 122 ± 68 μmol; with the use of the day-5 value for *Fa* × *S*, TBS were 122 ± 70 μmol. These values are not significantly different from the model-predicted population mean value for M_s_ [M(6)] of 120 ± 70 μmol.

**FIGURE 5:**
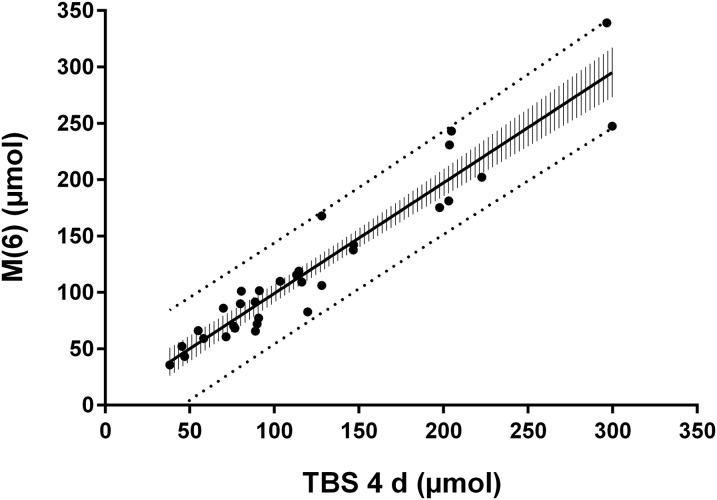
Model-predicted values for M(6) compared with TBS. Values for TBS were predicted by Equation *3*: TBS = *Fa* × *S* × (1/SA_p_), where the coefficients *Fa* and *S* were determined from the model (Figure 1) and 1/SA_p_, the specific activity of retinol in plasma, was determined analytically in plasma samples and then the model was used to calculate the value on day 4. The least-squares regression line was M(6) = 0.98 × TBS (4 d) + 1.12 (*r* = 0.95, *P* < 0.0001). Curved confidence bands are 95% CIs for the regression line, and straight prediction bands are 95% prediction intervals for individual subjects. *Fa*, the fraction of the orally administered dose of stable isotope–labeled vitamin A that was absorbed and retained; M(6), vitamin A mass in compartment 6; *S*, the ratio of specific activity of retinol in plasma to that in stores; SA_p_, plasma retinol specific activity; TBS, vitamin A total body stores.

Linear regression analysis also indicated that Equation *3*–derived values for TBS for individual subjects in this group were highly correlated with model-predicted values [*r* = 0.95 for day 4 data (**Figure 5**) and 0.96 for day 5 (data not shown), *P* < 0.001 for both analyses]. The least-squares lines (*y* = 0.98*x* + 1.12 for day 4 and *y* = 0.96*x* + 2.44 for day 5) were not significantly different from *y* = *x* (*P* = 0.74 for day 4 data and *P* = 0.49 for day 5). As indicated by the 95% CIs for the individual estimates shown in Figure 5 for day 4, values for TBS calculated by Equation *3* for a given individual were within ±20 μmol (1 SD) or 16% of model-predicted values for M(6). The rankings of TBS and M(6) for individuals were highly correlated, as indicated by Spearman rank correlation coefficients (*R*_s_ = 0.94, *P* < 0.001, for both times). The ratio of calculated (TBS) to model-predicted [M(6)] values ranged from 0.76 to 1.4 for the 32 individuals. In addition, the relative accuracy of the prediction [TBS/M(6)] was not significantly affected by the size of vitamin A body stores (*P* > 0.26).

To better understand why Equation *3* provided such a good estimate of mean and individual TBS, we investigated the contributions of the factors 1/SA_p_, *S*, and *Fa* in the prediction equation at 4 and 5 d. We used the compartmental model to estimate each variable for each subject on both days. When we plotted M(6), the model-predicted TBS, compared with 1/SA_p_ and fit the data to a linear equation (data not shown), the coefficient of determination (*R*^2^) was 0.91 at 4 d and 0.92 at 5 d; when *Fa* × *S* was included in the regression equation, the remaining variance was accounted for. In other words, >90% of the variation in M(6) was accounted for by the variance in 1/SA_p_ at 4 or 5 d; most of the remaining 8–9% was accounted for by variance in *S*. In contrast, at later times, the contributions of 1/SA_p_, *Fa*, and *S* changed, such that at 14 d, they were 72%, 25%, and 3%, respectively. In addition it is worth noting that, when we calculated TBS over time with the use of Equation *3* and the model-predicted values for *Fa*, *S*, and 1/SA_p_, TBS were constant.

## Discussion

In this study, we used results from model-based compartmental analysis of data on plasma [^13^C_10_]retinol kinetics to modify and improve the RID equation (“Olson equation” Equation *1*), which has been used to estimate vitamin A status and to evaluate the efficacy of vitamin A supplementation programs by numerous researchers over the past 25 y. Our goal was to determine whether a modified equation could accurately predict total body vitamin A stores in individual subjects as well as in groups by using data collected within 1 wk after administration of labeled vitamin A. In the original RID study (2), when TLR calculated by using Equation *1* was compared with extrapolated liver vitamin A concentrations based on biopsy, the ratio was 1.10 but values varied over a wide range (0.47–2.01; *n* = 10). When Equation *3* predictions are compared with model-predicted TBS in our study, agreement was excellent (ratio of 1.03) and the range was tighter (0.76–1.4; *n* = 32), so that predictions for individuals were very good.

In the original RID equation (2) (Equation *1* above), there was overlap in the definitions of factors *F* (the fraction of the orally administered dose of deuterated vitamin A that was absorbed and retained) and *a* (a correction for fraction of the absorbed dose lost via catabolism). We combined *F* and *a* into an aggregate factor, *Fa*, with the use of the following logic. The value for factor *a* in Equation *1* was calculated as follows:
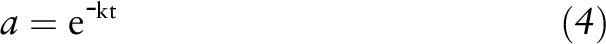
where *k* = (ln 2)/140 d [140 d is the estimated half-life (t_1/2_) of liver vitamin A] and *t* = days since the dose was administered. In fact, t_1/2_ will be dependent on vitamin A stores and will be much shorter in individuals with low stores. A careful review of the work upon which the original equation was based (4) showed that *a* is actually already taken into account in factor *F*. That is, because *F* was defined as the fraction of the dose that was absorbed and retained (i.e., was still in stores at the time of blood sampling), all loss due to degradation is incorporated into *F.* Thus, we combined factors *F* and *a* into factor *Fa.*

With regard to *Fa*, it is important to emphasize that this factor depends on, among other processes, vitamin A absorption efficiency. Currently, only limited information is available on the absorption efficiency of an oral dose of vitamin A in individual subjects. Thus, in future work, it will be important to determine absorption efficiency, especially if the goal is to obtain accurate values of TBS in individuals.

One of our objectives in the present work was to determine whether the original RID equation could be modified to use plasma isotope enrichment data from a blood sample collected earlier than at ∼21 d after dose administration as has been typically done (20, 21). Twenty-one days has been assumed to be optimal for tracer to thoroughly mix with body vitamin A pools (3, 22). However, researchers have suggested several potential benefits to earlier sampling, including improved subject availability and compliance and a lower likelihood of changes in health status during the test (e.g., development of infections). In addition, Adams and Green (23) reported that the optimal TBS prediction time in rats with a wide range of liver vitamin A concentrations was 3 d after dosing. The early sampling time improved sensitivity of the prediction, allowing for better discrimination between different levels of vitamin A status.

In view of these considerations, several researchers previously used early postdose data to predict vitamin A status by isotope dilution. For example, Ribaya-Mercado et al. (24) found that decreased plasma D:H ratios measured 3 d after dosing reflected improvements in vitamin A status in Filipino children who had received vitamin A supplementation; these researchers recommended the development of a 3-d prediction equation. Later, Tang et al. (25) measured D:H in serum 3 d after dosing, extrapolated that to a 21-d ratio, and used Equation *1* to predict TBS. In addition, Haskell et al. (26) used postequilibrium plasma isotope ratios and a modified Equation *1* to predict TBS in Peruvian children. These authors then developed a regression equation to predict TBS on the basis of the isotope ratio at 3 d. Finally, preliminary work with this data set (13) indicated that data from an early time (3 d) indeed held promise for estimating TBS. The current work further supports the benefit of using early data for determining both individual and mean TBS, because we found (Figure 2) that the mixing of tracer and tracee (interfusion) was complete by ∼1 wk after dose administration (i.e., much earlier than previously assumed).

In the original publication of the RID equation (2), values for the factors *F, a*, and *S* were assigned on the basis of previous research. Specifically, *F* was assigned a value of 0.5 on the basis of work in rats by Rietz et al. (4) and *S* was set at 0.65 on the basis of studies of the metabolism of vitamin A in rats as a function of liver vitamin A stores (27). Factor *a* would equal 0.98 at both 4 and 5 d (based on *k* = 0.005 in Equation *4*). Given these values, the product of factors *F*, *a*, and *S* in Equation *1* would be 0.32, compared with model-derived values of 0.48 on day 4 and 0.43 on day 5 (*Fa* = 0.61 and 0.58 on days 4 and 5, respectively, and *S* = 0.78 and 0.74).

Given the time dependence of these factors as shown in Figure 4A, our results show that there will be improved prediction of individual and group mean TBS if *Fa* × *S* is determined when variance in the coefficients is lowest (Figure 4B). We found that the interindividual variance in the aggregate factor *Fa* × *S* was lowest (15%) at 4–5 d after dose administration. In addition, the results shown in Figure 3A indicate that irreversible loss of vitamin A has less influence on interindividual differences in *Fa* at these earlier times and an increasing effect later [i.e., the curves for L(10,6) and *Fa* diverge with time]. Thus, predictions of TBS for individual subjects will be less affected by variance in the factor *Fa* if the method is applied at earlier sampling times. In addition, the prediction of M(6) (i.e., TBS) is most sensitive to variance in the measured variable (specific activity in plasma) at 4 or 5 d. Taken together, these results indicate that it is important to sample near 4 or 5 d, when variance in *Fa* × *S* has less influence in, and 1/SA_p_ is most sensitive to, the prediction of mean and individual TBS.

In conclusion, RID is an accurate and relatively simple technique for determining TBS of vitamin A in intervention and assessment trials in the field. Here we show that, with the use of data from a plasma sample collected 4 or 5 d after isotope administration, the equation TBS = *Fa* × *S* × (1/SA_p_) (Equation *3*) accurately predicts individual and mean vitamin A TBS in healthy young adults of European ancestry who had normal plasma retinol concentrations and moderate vitamin A intakes. Specifically, mean TBS predicted by Equation *3* (122 μmol) were within 2% of, and not significantly different from, the model-predicted value (120 μmol); for individual subjects, the estimates were within ±20 μmol (1 SD) of model predictions. The very simple equation TBS ≈ 0.5 × (1/SA_p_) [or TBS ≈ 0.5 × dose/(tracer:tracee ratio)], where 0.5 is approximately the product of *Fa* (0.61) and *S* (0.78), gives an adequate estimate of individual vitamin A TBS at 4 d. If researchers are able to estimate the population mean value for *Fa* × *S* at a time when variance in these factors is minimal (e.g., at 4 or 5 d after dosing), Equation *3* will predict TBS for an individual in that population with reasonable accuracy. If the values for factors *Fa* and *S* were determined for individuals over time by using model-based compartmental analysis or other methods, then TBS can be estimated at any time after tracer administration.

## Footnotes

8 Abbreviations used: FCR_sys_, system fractional catabolic rate; FD, fraction of the oral dose; L(I,J), fractional transfer coefficient; MS/MS, tandem mass spectrometry; RID, retinol isotope dilution; TBS, total body stores; TLR, total liver reserve.

## References

[1] Hunt JR, Durpad AV Introduction to symposium proceedings “Applying Vitamin A Isotope Dilution Techniques to Benefit Human Nutrition.” Int J Vitam Nutr Res 2014;84(Suppl 1):7–8. 25537100 10.1024/0300-9831/a000180

[2] Furr HC, Amedee-Manesme O, Clifford AJ, Bergen HR III, Jones AD, Anderson DP, Olson JA Vitamin A concentrations in liver determined by isotope dilution assay with tetradeuterated vitamin A and by biopsy in generally healthy adult humans. Am J Clin Nutr 1989;49:713–6. 2648799 10.1093/ajcn/49.4.713

[3] Bausch J, Rietz P Method for the assessment of vitamin A liver stores. Acta Vitaminol Enzymol 1977;31:99–112. 580694

[4] Rietz P, Wiss O, Weber F Metabolism of vitamin A and the determination of vitamin A status. Vitam Horm 1974;32:237–49. 4617401 10.1016/s0083-6729(08)60014-x

[5] Rietz P, Vuilleumier JP, Weber F, Wiss O Determination of the vitamin A body pool of rats by an isotopic dilution method. Experientia 1973;29:168–70. 4571216 10.1007/BF01945454

[6] Green MH, Green JB Vitamin A intake and status influence retinol balance, utilization and dynamics in rats. J Nutr 1994;124:2477–85. 16856330 10.1093/jn/124.12.477

[7] Furr HC, Green MH, Haskell M, Mokhtar N, Nestel P, Newton S, Ribaya-Mercado JD, Tang G, Tanumihardjo S, Wasantwisut E Stable isotope dilution techniques for assessing vitamin A status and bioefficacy of provitamin A carotenoids in humans. Public Health Nutr 2005;8:596–607. 16236189 10.1079/phn2004715

[8] Haskell MJ, Jamil KM, Peerson JM, Wahed MA, Brown KH The paired deuterated retinol dilution technique can be used to estimate the daily vitamin A intake required to maintain a targeted whole body vitamin A pool size in men. J Nutr 2011;141:428–32. 21248191 10.3945/jn.110.133124

[9] Lopez-Teros V, Quihui-Cota L, Mendez-Estrada RO, Grijalva-Haro MI, Esparza-Romero J, Valencia ME, Green MH, Tang G, Pacheco-Moreno BI, Tortoledo-Oritz O, Vitamin A-fortified milk increases total body vitamin A stores in Mexican preschoolers. J Nutr 2013;143:221–6. 23256139 10.3945/jn.112.165506

[10] Green MH, Green JB, Furr HC; Vitamin A Tracer Task Force. Analysis of stable isotope data to estimate vitamin A body stores. Vienna (Austria): International Atomic Energy Agency; 2008.

[11] Gannon BM, Tanumihardjo SA Comparisons among equations used for retinol isotope dilution in the assessment of total body stores and total liver reserves. J Nutr 2015;145:847–54. 25809683 10.3945/jn.114.208132 PMC6619684

[12] Haskell MJ, Handelman GJ, Peerson JM, Jones AD, Atai Rabbi M, Awal MA, Wahed MA, Mahalanabis D, Brown KH Assessment of vitamin A status by the deuterated-retinol-dilution technique and comparison with hepatic vitamin A concentration in Bangladeshi surgical patients. Am J Clin Nutr 1997;66:67–74. 9209171 10.1093/ajcn/66.1.67

[13] Green MH Evaluation of the “Olson Equation,” an isotope dilution method for estimating vitamin A stores. Int J Vitam Nutr Res 2014;84(Suppl 1):9–15. 25537101 10.1024/0300-9831/a000181

[14] Green MH, Ford JL, Oxley A, Green JB, Park H, Berry P, Boddy AV, Lietz G Plasma retinol kinetics and β-carotene bioefficacy are quantified by model-based compartmental analysis in healthy young adults with low vitamin A stores. J Nutr 2016;146:2129–36. 10.3945/jn.116.233486 PMC5037873 27511941

[15] Oxley A, Berry P, Taylor GA, Cowell J, Hall MJ, Hesketh J, Lietz G, Boddy AV An LC/MS/MS method for stable isotope dilution studies of β-carotene bioavailability, bioconversion, and vitamin A status in humans. J Lipid Res 2014;55:319–28. 24158962 10.1194/jlr.D040204 PMC3886671

[16] Wastney ME, Patterson BH, Linares OA, Greif PC, Boston RC WinSAAM. In: Investigating biological systems using modeling: strategies and software. San Diego (CA): Academic Press; 1999 p. 95–138.

[17] Cifelli CJ, Green JB, Green MH Use of model-based compartmental analysis to study vitamin A kinetics and metabolism. Vitam Horm 2007;75:161–95. 17368316 10.1016/S0083-6729(06)75007-5

[18] Green MH, Green JB Quantitative and conceptual contributions of mathematical modeling to current views on vitamin A metabolism, biochemistry, and nutrition. Adv Food Nutr Res 1996;40:3–24. 8858804 10.1016/s1043-4526(08)60018-2

[19] Cifelli CJ, Green JB, Wang Z, Yin S, Russell RM, Tang G, Green MH Kinetic analysis shows that vitamin A disposal rate in humans is positively correlated with vitamin A stores. J Nutr 2008;138:971–7. 18424609 10.1093/jn/138.5.971

[20] Ribaya-Mercado JD, Mazariegos M, Tang G, Romero-Abal ME, Mena I, Solomons NW, Russell RM Assessment of total body stores of vitamin A in Guatemalan elderly by the deuterated-retinol-dilution method. Am J Clin Nutr 1999;69:278–84. 9989693 10.1093/ajcn/69.2.278

[21] Ribaya-Mercado JD, Solon FS, Fermin LS, Perfecto CS, Solon JAA, Dolnikowski GG, Russell RM Dietary vitamin A intakes of Filipino elders with adequate or low liver vitamin A concentrations as assessed by the deuterated-retinol-dilution method: implications for dietary requirements. Am J Clin Nutr 2004;79:633–41. 15051608 10.1093/ajcn/79.4.633

[22] Haskell MJ, Islam MA, Handelman GJ, Peerson JM, Jones AD, Wahed MA, Mahalanabis D, Brown KH Plasma kinetics of an oral dose of [2H4]retinyl acetate in human subjects with estimated low or high total body stores of vitamin A. Am J Clin Nutr 1998;68:90–5. 9665101 10.1093/ajcn/68.1.90

[23] Adams WR, Green MH Prediction of liver vitamin A in rats by an oral isotope dilution technique. J Nutr 1994;124:1265–70. 8064375 10.1093/jn/124.8.1265

[24] Ribaya-Mercado JD, Solon FS, Solon MA, Cabal-Barza MA, Perfecto CS, Tang G, Solon JAA, Fjeld CR, Russell RM Bioconversion of plant carotenoids to vitamin A in Filipino school-aged children varies inversely with vitamin A status. Am J Clin Nutr 2000;72:455–65. 10919941 10.1093/ajcn/72.2.455

[25] Tang G, Qin J, Hao L-y, Yin S-a, Russell RM Use of a short-term isotope-dilution method for determining the vitamin A status of children. Am J Clin Nutr 2002;76:413–8. 12145015 10.1093/ajcn/76.2.413

[26] Haskell MJ, Lembcke JL, Salazar M, Green MH, Peerson JM, Brown KH Population-based plasma kinetics of an oral dose of [^2^H_4_]retinyl acetate among preschool-aged, Peruvian children. Am J Clin Nutr 2003;77:681–6. 12600861 10.1093/ajcn/77.3.681

[27] Hicks VA, Gunning DB, Olson JA Metabolism, plasma transport and biliary excretion of radioactive vitamin A and its metabolites as a function of liver reserves of vitamin A in the rat. J Nutr 1984;114:1327–33. 6737092 10.1093/jn/114.7.1327

